# A Simple and Universal System for Gene Manipulation in *Aspergillus fumigatus*: *In Vitro*-Assembled Cas9-Guide RNA Ribonucleoproteins Coupled with Microhomology Repair Templates

**DOI:** 10.1128/mSphere.00446-17

**Published:** 2017-11-22

**Authors:** Qusai Al Abdallah, Wenbo Ge, Jarrod R. Fortwendel

**Affiliations:** Department of Clinical Pharmacy and Translational Science, University of Tennessee Health Science Center, Memphis, Tennessee, USA; Carnegie Mellon University

**Keywords:** *Aspergillus fumigatus*, CRISPR-Cas9, gene deletion, genome editing, *in vitro* assembly, *pksP* gene

## Abstract

CRISPR (clustered regularly interspaced short palindromic repeat)-Cas9 is a novel genome-editing system that has been successfully established in *Aspergillus fumigatus*. However, the current state of the technology relies heavily on DNA-based expression cassettes for delivering Cas9 and the guide RNA (gRNA) to the cell. Therefore, the power of the technology is limited to strains that are engineered to express Cas9 and gRNA. To overcome such limitations, we developed a simple and universal CRISPR-Cas9 system for gene deletion that works across different genetic backgrounds of *A. fumigatus*. The system employs *in vitro* assembly of dual Cas9 ribonucleoproteins (RNPs) for targeted gene deletion. Additionally, our CRISPR-Cas9 system utilizes 35 to 50 bp of flanking regions for mediating homologous recombination at Cas9 double-strand breaks (DSBs). As a proof of concept, we first tested our system in the Δ*akuB* (Δ*akuB*^*ku80*^) laboratory strain and generated high rates (97%) of gene deletion using 2 µg of the repair template flanked by homology regions as short as 35 bp. Next, we inspected the portability of our system across other genetic backgrounds of *A. fumigatus*, namely, the wild-type strain Af293 and a clinical isolate, *A. fumigatus* DI15-102. In the Af293 strain, 2 µg of the repair template flanked by 35 and 50 bp of homology resulted in highly efficient gene deletion (46% and 74%, respectively) in comparison to classical gene replacement systems. Similar deletion efficiencies were also obtained in the clinical isolate DI15-102. Taken together, our data show that *in vitro*-assembled Cas9 RNPs coupled with microhomology repair templates are an efficient and universal system for gene manipulation in *A. fumigatus*.

**IMPORTANCE** Tackling the multifactorial nature of virulence and antifungal drug resistance in *A. fumigatus* requires the mechanistic interrogation of a multitude of genes, sometimes across multiple genetic backgrounds. Classical fungal gene replacement systems can be laborious and time-consuming and, in wild-type isolates, are impeded by low rates of homologous recombination. Our simple and universal CRISPR-Cas9 system for gene manipulation generates efficient gene targeting across different genetic backgrounds of *A. fumigatus*. We anticipate that our system will simplify genome editing in *A. fumigatus*, allowing for the generation of single- and multigene knockout libraries. In addition, our system will facilitate the delineation of virulence factors and antifungal drug resistance genes in different genetic backgrounds of *A. fumigatus*.

## Abstract (precis)

Tackling the multifactorial nature of virulence and antifungal drug resistance in *A. fumigatus* requires the mechanistic interrogation of a multitude of genes, sometimes across multiple genetic backgrounds. Classical fungal gene replacement systems can be laborious and time-consuming and, in wild-type isolates, are impeded by low rates of homologous recombination. Our simple and universal CRISPR-Cas9 system for gene manipulation generates efficient gene targeting across different genetic backgrounds of *A. fumigatus*. We anticipate that our system will simplify genome editing in *A. fumigatus*, allowing for the generation of single- and multigene knockout libraries. In addition, our system will facilitate the delineation of virulence factors and antifungal drug resistance genes in different genetic backgrounds of *A. fumigatus*.

## INTRODUCTION

**FIG 1:**
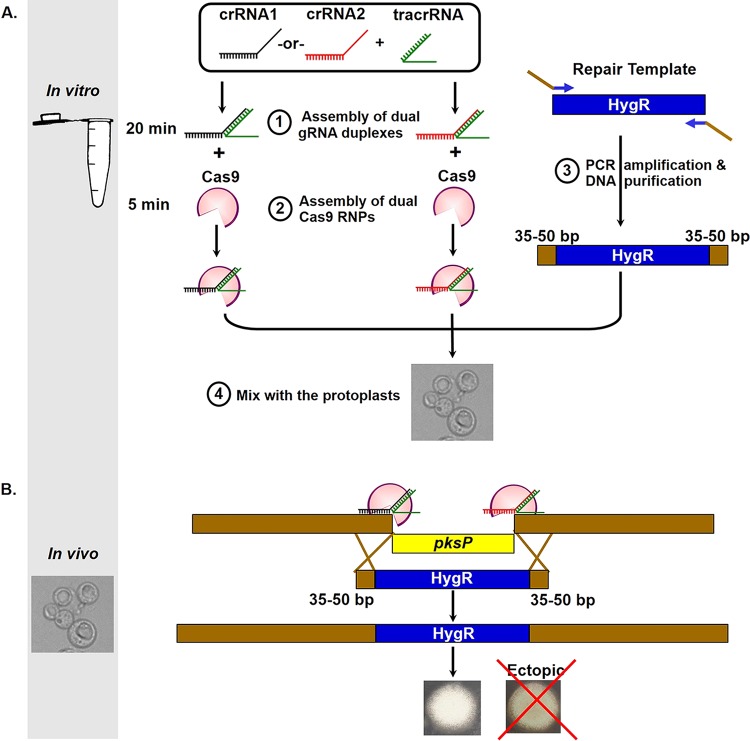
Overview of microhomology-mediated gene deletion coupled with *in vitro*-assembled dual Cas9 RNP cleavage. (A) (1) Cas9, tracrRNA, and dual crRNAs that cleave upstream and downstream of the *pksP* gene were purchased from a commercial vendor. The assembly of dual gRNA duplexes was performed by separately mixing each crRNA with equimolar amounts of tracrRNA to a final concentration of 33 μM. The two mixtures were boiled at 95°C for 5 min and then cooled to room temperature (20 to 25°C) for 10 to 15 min to allow hybridization of the crRNA to the tracrRNA. (2) For generation of dual Cas9 RNPs, each gRNA duplex was separately mixed with Cas9 (1 µg/µl) and incubated at room temperature for 5 min to allow for the formation of RNP complexes. (3) For generation of the repair template, the HygR cassette was PCR amplified using primer sets that insert 35 bp or 50 bp of flanking microhomology regions for targeting the *pksP* gene locus. The resulting PCR fragments were purified and utilized as repair templates. (4) The two RNP reaction mixtures were mixed with the HygR repair template and then added to *A. fumigatus* protoplast suspension (5 × 10^7^ conidia/ml). The protoplasts were then transformed according to a standard protocol. (B) Inside the protoplasts, the dual Cas9 RNPs cleave upstream and downstream of *pksP*, resulting in complete removal of the *pksP* coding sequence. In the presence of the HygR repair template, the cleaved *pksP* gene is replaced by the HygR repair template mediated by 35 to 50 bp of microhomology regions. Deletion mutants of the *pksP* gene exhibit white conidia, which allow for simple assessment of gene deletion based on the conidial color of the colonies.

CRISPR (clustered regularly interspaced short palindromic repeat)-Cas9 is an emerging tool for programmable genome editing in prokaryotes and eukaryotes (for reviews, refer to references 1 and 2. In this technique, which was originally discovered in bacteria as a mechanism of acquired resistance against viral infections, the Cas9 DNA nuclease recognizes and cleaves specific DNA sequences after forming a ribonucleoprotein (RNP) complex with a guide RNA (gRNA) (3–5). This gRNA is a duplex that is composed of a CRISPR RNA (crRNA) and a transactivating CRISPR RNA (tracrRNA) (1, 2). The crRNA contains a 20-base stretch designated the “protospacer,” which facilitates specific DNA cleavage by binding to complementary sequences in the target site (6). However, Cas9-mediated DNA cleavage occurs only if the protospacer is followed by a protospacer-adjacent motif (PAM) in the target sequence. For the commonly used Cas9 from *Streptococcus pyogenes*, the PAM site is normally an NGG (2, 7). Additionally, the crRNA contains a 22-base stretch for binding the tracrRNA (refer to Fig. 1 in reference 8. Although the tracrRNA does not directly mediate DNA binding processes, it is crucial for regulating crRNA maturation and DNA cleavage by crRNA-bound Cas9 (9, 10). In addition to the naturally occurring crRNA/tracrRNA duplex, single-guide-RNA (sgRNA) systems have been engineered to incorporate crRNA and tracrRNA into a single chimeric RNA (2, 9).

The DNA double-stranded break (DSB) introduced by Cas9 is primarily repaired by three pathways: nonhomologous end joining (NHEJ), homology-directed repair (HDR), and microhomology-mediated end joining (MMEJ). The NHEJ pathway (also termed classical nonhomologous end joining [C-NHEJ]) is used to introduce indels (i.e., insertion or deletions) with variable length at the DSB site, resulting in frameshift mutations. In contrast, the HDR pathway integrates an endogenous sister chromatid or an exogenous repair template containing large regions of DNA homologous to sequences flanking the DSB site. Therefore, the HDR pathway introduces mutations ranging from a 1-bp point mutation up to the integration of several coding sequences at the DSB site (2, 11). In the MMEJ pathway, which is a subset of the alternative nonhomologous end joining pathway (A-NHEJ), short homology regions (i.e., microhomology) flanking the upstream and downstream regions of the DSB site are recombined to restore DNA integrity (see Fig. 1 in references 12 and 13. With the widespread use of CRISPR-Cas9 for genome editing, the MMEJ pathway has also now been employed for Cas9-mediated manipulations by using exogenous repair templates containing microhomology regions flanking the DSB site (11).

Although the HDR and MMEJ repair machineries are both activated by a DSB and require a linear DNA fragment for DSB repair, each pathway retains distinct mechanisms for processing the resected ends of the DSB (for reviews on the mechanisms of HDR and MMEJ repair pathways, refer to reference 14. HDR is an error-free repair pathway which is predominantly active during the late S phase and the entire G_2_ phase of the cell cycle. In contrast, MMEJ is an error-prone pathway that is activated during G_1_ and early S phases of the cell cycle (12, 14–16). Another major difference between HDR and MMEJ is the size of the homology regions harbored within the linear DNA fragment. Whereas HDR requires long homology flanking regions (~500 to 5,000 bp) for precise insertion of the repair template, MMEJ-based integration of the repair template is mediated by short microhomology regions (2 to 40 bp) (12, 17–19).

*Aspergillus fumigatus* is the most common etiologic agent of invasive aspergillosis (IA), an often-fatal infection in immunocompromised patients (20, 21). Although current antifungals display anti-*Aspergillus* activity, treatment of IA infections is being challenged by the emergence of antifungal drug resistance (22). Therefore, there is an increasing need for the development of efficient genome-editing platforms to identify virulence factors and antifungal drug resistance factors across genetically distinct strains of *A. fumigatus*. Several CRISPR-Cas9 platforms have been established successfully to edit the genome of *A. fumigatus* (23–26). However, the current state of the technology relies heavily on DNA-based systems for delivering Cas9 and the gRNA to the nucleus. In these systems, Cas9 and gRNA expression cassettes are delivered on linear DNA fragments or circular plasmids to the nucleus. After integration in the genome, Cas9 and the gRNA are produced to form a Cas9 RNP complex (23–26). Alternatively, transient CRISPR-Cas9 expression systems that utilize autonomously replicating plasmids were also established in *A. fumigatus* (24). The major limitation to such DNA-based systems is that they require the construction of expression vectors and fungal strains for their use, restricting studies to these strains only. Additionally, DNA-based systems require the construction of species-specific expression cassettes. However, this might challenge the portability of CRISPR-Cas9 to other fungi that lack well-established genetic tools (27).

One method to overcome these drawbacks is to use *in vitro*-assembled Cas9 RNPs. In this system, Cas9 and gRNA expression cassettes are replaced by purified Cas9 enzyme and synthetic gRNA that are either purchased from commercial vendors or produced in the laboratory. Cas9 and the gRNA are mixed *in vitro* to allow the formation of functional RNPs, which are then mixed with a repair template and delivered into the host cell using traditional transformation procedures. *In vitro* assembly of Cas9 RNPs has been implemented previously for genome editing of *Penicillium chrysogenum* and several *Candida* species (27, 28). The major advantages of this system are simplicity (i.e., it does not require strain construction) and portability (it can be used across different species of the same genus or different strains of the same species). Accordingly, we sought in this work to utilize the advantages of the *in vitro* assembly of Cas9 RNPs to develop a universal system for gene deletion that works across different genetic backgrounds of *A. fumigatus*. Our system utilizes dual Cas9 RNP complexes that cleave upstream and downstream of the target gene, allowing for complete deletion of the target gene. Deleted genes are then replaced by a repair template (i.e., an antibiotic selection cassette) that is flanked by 35 to 50 bp of microhomology regions adjacent to Cas9 cutting sites (Fig. 1). Our results suggest that this system is a simple yet efficient tool for manipulating genomes of different *A. fumigatus* laboratory strains and clinical isolates.

## RESULTS

### Selection of the crRNA protospacer sequences for complete deletion of the *pksP* gene.

Our goal was to establish a simple and universal system for complete gene deletion that coupled *in vitro* assembly of dual Cas9 RNPs with a repair template that is flanked by microhomology regions adjacent to the target gene. Gene replacement efficiency was evaluated by using the polyketide synthase gene (*pksP*, Afu2g17600) as a target gene throughout this study. PksP mediates the biosynthesis of dihydroxynaphthalene (DHN)-melanin, a secondary metabolite that accounts for the gray-green color of conidia in *A. fumigatus* (29). Deletion of the *pksP* gene in *A. fumigatus* results in white conidia, allowing for simple assessment of gene deletion efficacy based on the conidial color of the resulting transformants. The *pksP* gene has been previously used as a reporter gene to assess the efficacy of several CRISPR-Cas9 platforms in *A. fumigatus* (23, 24).

**FIG 2:**
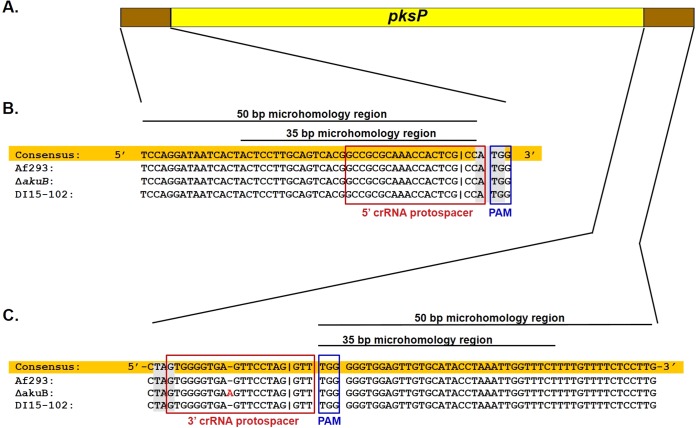
Selection of dual crRNA protospacer sequences. (A) Schematic diagram of *pksP* coding sequence and the flanking regions that are targeted by dual *in vitro*-assembled Cas9 RNPs. Designing the protospacers is described in Results. (B and C) Sequence alignment of *pksP* upstream (B) and downstream (C) regions of three distinct genetic backgrounds of *A. fumigatus*. The consensus sequence (highlighted in orange) was manually generated based on sequence alignment. The 5′ crRNA and 3′ crRNA protospacer sequences are marked by red open boxes. The protospacer-adjacent motif (PAM) sequences are marked by blue open boxes. Start and stop codons are highlighted in gray. The additional adenine in the *pksP* downstream region of the Δ*akuB* strain and the clinical isolate DI15-102 is shown in red font. Cas9 DSB sites (i.e., 3 nucleotides upstream of the PAM site [1, 9, 57]) are marked by a vertical line in the sequence. The sequences of the 35-bp and 50-bp regions that are used for microhomology-mediated integration are marked by a line above the sequence.

To delete the entire coding sequence of *pksP*, we designed two crRNAs (designated cr5′pksP and cr3′pksP) that direct Cas9 cleavage within the 5′ untranslated region (UTR) and 3′ UTR of *pksP*, respectively (Fig. 2B and C; Table 1). We used the well-annotated reference strain Af293 to design the crRNAs. We first identified all PAM sites (NGG) located from −30 to +4 bp upstream and from −30 to 0 bp downstream of the *pksP* start and stop codons, respectively. Two PAM sites were found in the *pksP* upstream region: one located within the first 4 nucleotides of the *pksP* coding sequence and the second located 18 bp upstream of the *pksP* start codon. To limit the deletion of non-*pksP* coding sequences, we chose the cr5′pksP protospacer with the closest proximity to the start codon (Fig. 2B). The 30-bp region downstream of the *pksP* gene was noted to be G rich, and thus, several PAM sites were identified (Fig. 2C). The cr3′pksP protospacer was chosen because it precedes a multiple-PAM-site region (Fig. 2C) and such regions have been shown to increase Cas9 cleavage specificity (30).

**TABLE 1:** Oligonucleotides and crRNAs used for this study

Type, purpose, and name	Sequence^a^
Oligonucleotides for	
Cloning HygR cassette into pCR-Blunt II-TOPO	
gpdA(p)-For	CGAGCTCCCAAATCTGTCC
trpC(t)-Rev	AGCTTGCATGCCTGCAGG
Amplification of HygR cassette with 35-bp microhomology	
35 bp-pksP-HygR-F	ACTCCTTGCAGTCACGGCCGCGCAAACCACTCGCC**CGAGCTCCCAAATCTGTCCA**
35 bp-pksP-HygR-R	AGAAACCAATTTAGGTATGCACAACTCCACCCCCA**AGCTTGCATGCCTGCAGGTC**
** **Amplification of HygR cassette with 50-bp microhomology	
50 bp-pksP-HygR-F	TCCAGGATAATCACTACTCCTTGCAGTCACGGCCGCGCAAACCACTCGCC**CGAGCTCCCAAATCTGTCCA**
50 bp-pksP-HygR-R	CAAGGAGAAAACAAAAGAAACCAATTTAGGTATGCACAACTCCACCCCCA**AGCTTGCATGCCTGCAGGTC**
** **Amplification of Southern blot probe	
pksP-Probe-Forward	GGCACGCTGTGCAGGACG
pksP-Probe-Reverse	GCCCGTCATCCCACTTGC
** **Sequencing DI15-102 isolate	
pksP(p)-159-for	TGAAGAGGTTGGACCCCAGTCGT
pksP-141-Rev	TTCCTGACGCAGTGCATGGAAGC
pksP-6521-For	CCAAACAAATGGGATACTTTGGTGGGCC
pksP(t)-159-Rev	ATTAGATACTCCAAACGCAACTAACTTCGGGTTCA
Seq-pksP(p)-126-For	GCACTTAAAGACTCCTCTTTTCAA
Seq-pksP-6606_For	CGGGACAGAAGGCGAA
crRNA sequences	
cr5′pksP	GCCGCGCAAACCACTCGCCA
cr3′pksP	GTGGGGTGAGTTCCTAGGTT

a Underlined sequences are the microhomology arms; bold sequences are for the amplification of the HygR cassette.

The chosen protospacers were then examined for sequence conservation in the Δ*akuB*^*ku80*^ strain (henceforth referred to as the Δ*akuB* strain) and DI15-102. Because the genome sequence of the DI15-102 clinical isolate is not available, we used Sanger technology to sequence the 50-bp and 69-bp regions that are located upstream and downstream of the *pksP* start and stop codons, respectively. Next, we manually carried out sequence alignment for the 50-bp and 69-bp regions among Af293, the Δ*akuB* strain, and the clinical isolate. With the exception of an adenine located 9 bases downstream of the stop codon in the Δ*akuB* strain, our data demonstrate that the two regions are conserved among all three strains of *A. fumigatus* (Fig. 2A to C). Although the target sequence of the cr3′pksP protospacer contains an additional adenine in the Δ*akuB* strain, this additional adenine is located outside the last 6 bp of the protospacer, known as the seed region (Fig. 2C). While mismatches in the seed region have been shown to abrogate Cas9 cleavage activities, mismatches outside this region do not significantly reduce Cas9 cleavage activities (31).

Finally, each protospacer sequence was utilized to perform a BLAST search in the *A. fumigatus* Af293 genome (http://www.aspgd.org). To minimize Cas9 off-target cleavage, protospacers that displayed greater than 15 bp of identity to off-target (yet PAM-adjacent) genomic loci should be omitted (32). Our BLAST analysis demonstrated that neither of the designed crRNAs displayed 15-bp (or more) off-target identity in the *A. fumigatus* genome (data not shown). Additionally, off-target sequences with high homology to either of the designed crRNAs were lacking adjacent PAM sites, which are essential for Cas9 recognition of target sequences (9, 33–35). Accordingly, we decided to exploit these two regions for Cas9 cleavage and gene replacement events.

### *In vitro*-assembled dual Cas9 RNPs coupled with microhomology repair templates result in efficient gene targeting in various *A. fumigatus* genetic backgrounds. (i) The *A. fumigatus* Δ*akuB* strain, a laboratory strain with a defective NHEJ pathway.

**FIG 3:**
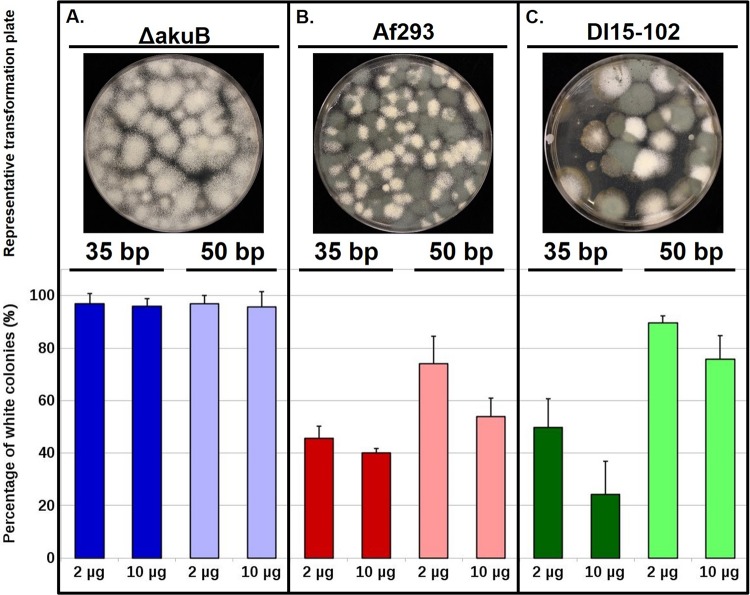
High efficiency of gene deletion in all tested genetic backgrounds of *A. fumigatus*. *In vitro*-assembled Cas9 RNPs coupled with microhomology-mediated integration of the HygR cassette were tested in Δ*akuB* (A), Af293 (B), and DI15-102 (C) strains. (Above) Representative transformation plates are shown for each strain using 2 µg of the HygR repair template that is flanked by 35-bp microhomology arms. (Below) The assessment of *pksP* deletion efficiency across different strains is plotted as the number of Δ*pksP* mutants out of the total number of transformation colonies. Deletion efficiencies were assessed based on the color of conidia. The Δ*pksP* mutant produces white colonies, while ectopic integrations result in green colonies. Deletion efficiencies represent the average from at least three independent transformations. Error bars represent the standard deviation calculated for each combination of strain, the size of HygR microhomology arms, and concentration of the HygR repair template for all experimental replicates.

As a proof of concept, our initial experimentation focused on deletion of *pksP* in the Δ*akuB* strain. This strain is deficient in NHEJ repair and thus exhibits high rates of homologous recombination (36). This strain has been an important tool for *A. fumigatus* genetics research as a method for circumventing the typically low level of homologous recombination in wild-type strains. The repair template employed for gene replacement included a hygromycin resistance cassette (HygR), allowing selection of transformants resistant to hygromycin. To analyze the importance of the length of the homology arms flanking the HygR cassette, we investigated the effect of two sizes of microhomology regions on the efficiency of gene replacement. Thus, oligonucleotides designed for amplification of the HygR repair template included two components: (i) a 20-base region of homology to the HygR cassette for template annealing during PCR amplification and (ii) a region of microhomology of either 35 or 50 bp in length that was targeted to the genomic DNA sequence flanking the Cas9-induced DSBs at the *pksP* locus (Table 1; Fig. 2B and C). Finally, we also examined the effect of repair template concentration on gene replacement efficiency by performing separate transformation experiments with two different concentrations of repair template DNA (i.e., 2 µg or 10 µg). The *in vitro* assembly of Cas9 RNPs and subsequent transformation of *A. fumigatus* were both performed as described in the legend to Fig. 1 and in Materials and Methods. Gene replacement efficiency, calculated as the percentage of white colonies among the total number of transformants, was close to 100% in all four experiments using the Δ*akuB* strain as the parent strain. The gene replacement efficiency was approximately 97% using 2 µg of either 35-bp or 50-bp microhomology repair templates (Fig. 3A). When we increased the amount of the repair template to 10 µg, we did not observe a significant change in gene replacement efficiency (Fig. 3A). Moreover, the additional adenine in the *pksP* downstream region of the Δ*akuB* strain (Fig. 2C) did not seem to affect cr3′pksP binding activities and Cas9 cleavage since *pksP* deletion efficiencies in this strain were close to 100% (Fig. 3A). Taken together, our results demonstrate that *in vitro*-assembled Cas9 RNPs coupled with repair templates harboring as little as 35 bp of sequence homology are sufficient to generate high rates of gene deletion in the NHEJ-deficient strain. In this genetic background, the concentration of the HygR repair template did not appear to affect homologous recombination rates.

### (ii) *A. fumigatus* strain Af293, the reference genome strain. 

**FIG 4:**
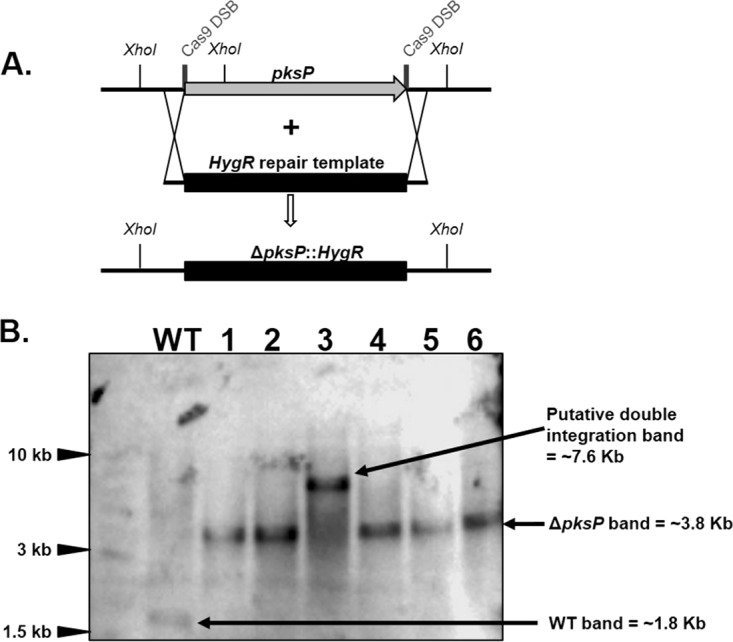
Southern blot analysis of Δ*pksP* mutant generated in the Af293 background. (A) Schematic representation of the genomic locus of the Af293 and Δ*pksP* strains. Deletion of the *pksP* gene was carried out using the HygR cassette. The cleavage sites of the dual *in vitro*-assembled Cas9 RNPs are marked by thick vertical lines. XhoI cutting sites are indicated in the *pksP* locus of the wild-type and Δ*pksP* strains. (B) Southern blot analysis of 6 arbitrarily selected colonies after digesting genomic DNA with the XhoI restriction enzyme. The wild type (WT) produced a 1.8-kb band that matches the expected wild-type banding pattern. Lanes 1, 2, 4, 5, and 6 displayed a 3.8-kb band which matches the expected *pksP* deletion banding pattern. The colony in lane 3 displayed a 7.6-kb band, likely containing a tandem integration of the HygR repair template at the *pksP* locus.

Interstrain variability of virulence is a recently recognized and important phenomenon among environmental fungal pathogens like *A. fumigatus*. In fact, recent studies have highlighted important differences in virulence attributes between the two major laboratory strains of *A. fumigatus*, Af293 and CEA10 (37). Therefore, it is important to develop tools that may facilitate molecular studies across multiple *A. fumigatus* strains. Because the Δ*akuB* mutant was generated in the CEA10 genetic background (36), we sought to test our system in the *A. fumigatus* reference strain Af293. In contrast to the Δ*akuB* strain, the Af293 wild-type strain displays low levels of homologous integration using standard transformation protocols. Nevertheless, using our portable CRISPR-Cas9 system, we were able to increase gene deletion frequencies to 40 to 74% in Af293. When transformations were performed with 2 µg of the HygR repair template and 35-bp-homology flanking regions, 46% (±4.8%) *pksP* gene replacement efficiencies were observed (Fig. 3B). When the length of the homology flanks was increased to 50 bp, *pksP* gene deletion efficiencies increased to 74% (±10.5%) (Fig. 3B). However, when the amount of the repair template was increased to 10 µg, gene deletion efficiencies dropped for 35-bp- and 50-bp-flanked HygR cassettes to 40% (±1.5%) and 54% (±6.9%), respectively (Fig. 3B). These data suggested that increasing the concentration of the repair template in a strain with an intact NHEJ pathway resulted in an increased number of ectopic integration events. We reasoned that, because Af293 has a functional NHEJ pathway, there was an increased chance of generating unwanted ectopic integrations even in the transformants counted as successful homologous recombination events (all white colonies). Therefore, we performed a Southern blot analysis using six randomly chosen white colonies from the Af293 transformations utilizing 2 µg of the transforming repair template and 35-bp microhomology. Our analysis demonstrated that five colonies displayed a single integration event with the expected *pksP* deletion band size (~3.8 kb) (Fig. 4A and B). One transformant, however, displayed a band at twice the predicted *pksP* deletion band size (~7.6 kb) while not producing the proper 3.8-kb *pksP* deletion band (Fig. 4A and B). In addition, this transformant did not produce a band matching the expected wild-type banding pattern (~1.8 kb). For these reasons, we presume that this transformant is likely to contain a tandem integration of the HygR repair template at the *pksP* locus. Interestingly, Southern blot analysis of six randomly chosen transformants from the Δ*akuB* strain experiment yielded the exact same results (data not shown). Although the downstream region of the *pksP* gene completely matches the cr3′pksP sequence (Fig. 2C), deletion efficiencies in the Af293 strain were lower than those obtained in the Δ*akuB* strain (Fig. 3A and B), which contains an additional adenine in the same region. These results suggest a major role for the DNA repair pathways in modulating repair template integration. In contrast, mutations outside the protospacer seed region exhibit minor effects on the efficiencies of gene deletion. Taken together, our data suggest that the typically low levels of site-specific integration in the Af293 wild-type strain can be overcome by coupling Cas9-mediated DSBs with a microhomology repair template. However, a small portion of the resulting transformants may carry tandem integrations at the target site.

### (iii) *A. fumigatus* DI15-102, a clinical isolate with an unknown genotype. 

With the emergence of antifungal drug resistance in *A. fumigatus*, direct manipulation of clinical isolates is currently receiving increased attention to delineate specific resistance mechanisms in diverse clinical isolate backgrounds (38). However, like the Af293 strain employed here, mutational analyses of *A. fumigatus* clinical isolates are typically plagued by the same low rates of homologous recombination. Therefore, we sought to test the portability of our system into an *A. fumigatus* clinical isolate with a largely uncharacterized genetic background. The *A. fumigatus* clinical isolate DI15-102 was employed for this purpose. DI15-102 has previously been confirmed as *A. fumigatus* by sequence analysis of the β-tubulin gene and has been characterized as triazole resistant (39). Employing DI15-102 as the parental strain, transformation with 2 µg of a HygR repair template that includes 35 bp of microhomology resulted in 50% (±11%) deletion efficiency of *pksP* (Fig. 3C). Increasing the length of the homology regions to 50 bp improved gene targeting rates to 90% (±2.7%). Similarly to the Af293 reference strain, increasing the concentration of the HygR repair template to 10 µg resulted in lower efficiency of gene targeting (Fig. 3C). Our data again suggested that increasing the concentration of the repair template from 2 µg to 10 µg resulted in an elevated number of nonspecific, ectopic integrations. Additionally, *pksP* deletion efficiencies in DI15-102 were comparable to or higher than those obtained in Af293 (Fig. 3B and C). Both strains exhibit a functional NHEJ repair pathway, and the *pksP* downstream region completely matches the cr3′pksP sequence (Fig. 2C). These results suggest that Cas9-mediated gene deletion is primarily controlled by DNA repair pathways and that the effect of mutations outside the protospacer seed region is minor. It is noteworthy that transformation efficiencies of the DI15-102 clinical isolate were significantly lower than those of the Δ*akuB* and Af293 strains, as significantly lower total numbers of transformants were obtained. However, variability in the sequence identity of upstream and downstream regions of homology is not the reason for decreased efficiency in the clinical isolate, as these areas are identical (Fig. 2B and C). Collectively, our data demonstrate that *in vitro*-assembled Cas9 RNPs coupled with microhomology repair templates are a simple and universal system that works in different genetic backgrounds of *A. fumigatus*.

### Concentration of Cas9 directly correlates with the efficiency of gene deletion.

**FIG 5:**
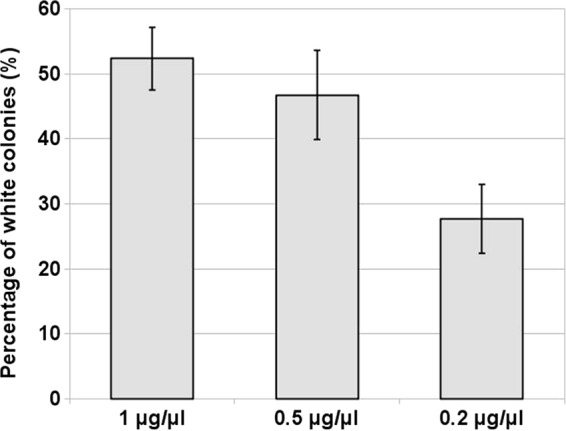
The concentration of Cas9 directly correlates with the efficiency of gene deletion. The analysis was carried out in the Af293 strain using 2 µg of the HygR repair template flanked by 35 bp of microhomology regions. Dilution of Cas9 is described in Materials and Methods. The effect of Cas9 concentration on *pksP* deletion rates was assessed based on the color of conidia. Deletion efficiencies represent the average from at least three independent transformations. Error bars represent the standard deviation calculated for each concentration of Cas9.

In the previous experiments, we analyzed the effects of the concentration of the repair template, the size of the flanking homology regions, and the genetic background of *A. fumigatus*. We found that the efficiencies of gene deletion are primarily controlled by DNA repair pathways. Additionally, our data demonstrate that increasing the length of the homology arms to 50 bp improved gene targeting rates in Af293 and DI15-102. In contrast, increasing the concentration of the HygR repair template to 10 µg reduced deletion efficiencies in Af293 and DI15-102, as increased numbers of ectopic integrations are obtained. Throughout these initial studies, we used Cas9 nuclease at a high concentration of 1 µg/µl. Therefore, we sought here to inspect the effects of Cas9 concentration on gene deletion efficiency. We analyzed gene targeting efficiencies at two additional concentrations of Cas9. Using 2 µg of the transforming HygR repair template with 35-bp microhomology regions, gene replacement efficacies were compared between 1-µg/µl, 0.5-µg/µl, and 0.2-µg/µl Cas9 concentrations in the Af293 wild-type strain. Transformations with 1 µg/µl of Cas9 resulted in 52% (±4.8%) white colonies (Fig. 5), a percentage that is comparable to our previous results with Af293 under the same conditions (Fig. 3B). Interestingly, when the Cas9 concentration was decreased to 0.5 µg/µl, *pksP* deletion efficiency was not significantly affected (Fig. 5); however, the overall transformation efficiency (i.e., total number of hygromycin-resistant transformants) decreased by ~60% (data not shown). In contrast, dilution of Cas9 to 0.2 µg/µl resulted in moderately reduced *pksP* deletion efficiency to 28% (±5.3%) and overall transformation efficacy by ~66%, respectively. Taken together, our data demonstrate that the concentration of Cas9 correlates with transformation and gene deletion efficiencies.

## DISCUSSION

The novelty of this work lies in establishing a universal platform for site-specific gene targeting that can be employed across various genetic backgrounds of *A. fumigatus*. This technique offers multiple advantages over the classical technologies, which rely on homologous recombination for DNA integration. First, our system is simple as it relies only on the *in vitro* assembly of commercially available Cas9 proteins with the appropriate crRNA and tracrRNA (also produced commercially) to form the necessary ribonucleoprotein complexes for gene targeting. Another major advantage of our platform is that it requires only microhomology regions (35 to 50 bp) for efficient gene deletion. Currently, classical gene deletion systems in *A. fumigatus* wild-type strains are dependent on laborious and time-consuming construction of selectable markers with large flanking homology regions (~750 to 1,500 bp) (40). We were able to omit the standard construct building steps of subcloning DNA fragments and/or overlap extension PCR and replace them with a single PCR that amplifies a selectable marker with incorporated microhomology regions. This repair template is then mixed with *in vitro*-assembled Cas9 RNPs and added to protoplasts according to standard transformation protocols to generate mutant strains. A third major advantage of our system is that it results in higher gene targeting rates than the classical systems. In the Af293 wild-type strain, we achieved 46% and 74% gene deletion rates using 35-bp and 50-bp homology regions, respectively. In contrast, classical gene manipulation systems normally result in very low (~5 to 20%) gene targeting rates in the Af293 strain.

Using our current protocol with commercially supplied components, we calculate an average cost of ~$165/transformation. One possibility to reduce the cost of *in vitro*-assembled Cas9 RNP systems is to produce purified Cas9 and gRNA components in the laboratory. Further, as with other innovations, we would expect the cost of commercially prepared Cas9 protein and the synthesis of tracrRNA and crRNA to drop over time. CRISPR-Cas9 systems have been previously established in *A. fumigatus* using DNA-based expression cassettes and are promoted as cheaper ways to employ CRISPR-mediated mutagenesis (23–25). However, employing DNA-based systems for genome editing has several disadvantages compared with the purified Cas9 RNP systems. First, the DNA-based systems can be laborious and time-consuming since they require the construction of vectors with different gRNAs for targeting different genes. Second, the portability of DNA-based systems can be challenging, as many fungal species lack well-established genetic tools (27). Third, there are risks that Cas9 and gRNA expression cassettes might integrate randomly in the genome or at the DSB site (23). Finally, DNA-based delivery systems can be associated with higher rates of off-target cleavage (discussed below).

To minimize the potential for off-site targeting in our system, we performed a BLAST analysis for each of the designed gRNAs. In order for Cas9 to bind and cleave off-target sites, two gRNA criteria must be met. The first criterion is the presence of ≤5 mismatches between the chosen gRNA sequence and the off-target sequence. Cas9 normally tolerates mismatches that are located at the 5′ end of the gRNA, outside the seed region (31). This is supported in our studies, as we found that a single nucleotide mutation located outside the crRNA seed region did not affect the portability of our *in vitro*-assembled Cas9 RNP system. The second criterion is that the off-target sequences must precede a PAM site (9, 33–35). Although our analysis identified several sequences that display 15 bases or less of identity to either of the gRNAs, these sequences were not followed by a PAM site and therefore were not considered potential Cas9 off-target sites. Further, the delivery of a purified Cas9 RNP complex has been associated with minimal off-target cleavage events (41–43). In contrast, DNA-based delivery systems could be complicated by the potential for higher numbers of off-target effects from Cas9 expression in the cell (44). For example, the use of regulatable promoter systems could lead to low levels of constitutively expressed Cas9 nuclease if induction or repression of expression is not tightly regulated. This, in turn, could lead to increased off-target DSBs and result in undesired phenotypes. Because our system relies on only transient presence of the Cas9 protein immediately following transformation, we believe that our *in vitro*-assembled Cas9-gRNA-based gene manipulation in *A. fumigatus* carries a relatively low risk for off-site mutations compared to DNA-based systems. Our laboratory is currently working to test this hypothesis.

Our study is the first report to apply an *in vitro* assembly of a CRISPR-Cas9 system for complete gene deletion in *A. fumigatus*. To achieve the highest possible gene deletion efficiencies, we implemented dual gRNAs to target both upstream and downstream PAM sites of the *pksP* gene. In contrast, previous studies have used only one gRNA for site-specific gene disruption via targeting sequences with close proximity to the annotated ATG (23, 24). However, such approaches have two major drawbacks. First, inaccuracies in genome annotation might cause targeting of a nearby upstream noncoding ATG instead of the actual start codon of the target gene (45, 46). Such an error might lead to the disruption of noncoding sequences instead of genes of interest. Second, alternative transcription and splicing are common in eukaryotes and produce multiple isoforms from the same gene. These various isoforms can then have different cellular functions (47). Gene disruption by insertion of selectable markers in a single site breaks the open reading frame of some gene isoforms but not others. To overcome these risks, complete gene deletion remains the most powerful tool for the analysis of gene function. Apart from this, our work also shows that targeting two separate PAM sites in a single transformation is an efficient process with *in vitro*-assembled Cas9 RNPs. This might open the door for several applications. For example, targeting two separate genes with fluorophore tags could be accomplished in a single experiment. Additionally, delineating multifactorial virulence factors in fungi requires the deletion of multiple genes in one fungal strain. Accordingly, our system might be useful for marker recycling. This process, which is designated CRISPR-Cas9-induced marker excision (CRIME), has been established successfully in *Candida albicans* using DNA-based CRISPR-Cas9 expression cassettes (48). CRIME is also feasible using *in vitro*-assembled dual Cas9 RNPs.

One prerequisite for our system is the availability of a PAM site on both ends of the target gene. This should be achievable in fungi with relatively high genomic GC content such as *A. fumigatus* (GC content is 50% [49]). However, the identification of two PAM sites flanking the target gene in close proximity to the start and stop codons remains a challenge in fungi like *Candida albicans*, which has a low genomic GC content (33.5%) (50). In these organisms, two alternative solutions can be applied. The first solution is to use the Cas9 homologue Cpf1, which targets AT-rich PAM sites. Cpf1 is a type V-A endonuclease of the CRISPR-Cas class 2 that recognizes a TTN PAM sequence, and therefore, it is suitable for editing genomes with a low GC content (51, 52). The second alternative is to generate oligonucleotides with large flanking regions that would, in effect, reincorporate areas of DNA removed by inducing DSBs at PAM sites distal to the stop or start codons.

In summary, we have developed a simple and universal gene manipulation system for *A. fumigatus* by combining the techniques of Cas9-gRNA *in vitro* assembly with a microhomology repair template and adapting them for *Aspergillus* transformation. This system allows for efficient gene targeting in multiple genetic backgrounds, including clinical isolates. Future studies will evaluate the potential for off-target DNA cleavage caused by our *in vitro* RNP system.

## MATERIALS AND METHODS

### Strains and preparation of conidia.

The wild-type strain *A. fumigatus* Af293, the clinical isolate DI15-102, and the genetically engineered Δ*akuB* (Δ*akuB*^*ku80*^) strain, which is deficient in the NHEJ pathway, were used in this study (36, 39, 53). For harvesting of conidia, all strains were cultivated on glucose minimal medium (GMM) agar plates (54) for 3 days at 37°C. Conidia were harvested from GMM agar plates using sterile water. After being washed three times with sterile water, conidia were counted and stored at 4°C until used.

### Construction and amplification of the HygR repair template.

A hygromycin B phosphotransferase expression cassette, referred to as here as HygR, was used as the selectable marker for Cas9-mediated gene deletion throughout this work. A 2,890-bp region, which spans 1,053 bp of the *gpdA* promoter, 1,020 bp of hygromycin B phosphotransferase (*hph*), and 817 bp of the *trpC* terminator, was PCR amplified from plasmid pUCGH (55) using the primers gpdA(p)-For and trpC(t)-Rev (Table 1). The resulting HygR cassette was cloned into pCR-Blunt II-TOPO using the Zero Blunt TOPO PCR cloning kit (Invitrogen) according to the manufacturer’s instructions. Positive clones were Sanger sequenced to confirm the absence of mutations, and the resulting plasmid was designated pCR-HygR. For generation of the repair templates needed for Cas9-mediated gene deletion, the HygR cassette was PCR amplified from plasmid pCR-HygR using either primer set 35 bp-pksP-HygR-F and 35 bp-pksP-HygR-R or primer set 50 bp-pksP-HygR-F and 50 bp-pksP-HygR-R. The resulting PCR fragments were purified using the GeneJET gel extraction kit (Thermo Scientific) and eluted using nuclease-free water. These purified PCR products were utilized as the completed repair templates and were composed of a 2,890-bp HygR cassette flanked by either 35 bp or 50 bp of microhomology regions targeting the *pksP* gene locus (Afu2g17600).

### *In vitro* assembly of Cas9-gRNA ribonucleoprotein complexes.

Cas9 ribonucleoproteins (RNPs), composed of crRNA, tracrRNA, and the Cas9 protein, were assembled *in vitro* using commercially available Alt-R-CRISPR-Cas9 components from Integrated DNA Technologies (IDT). For preparation of the synthesized RNA components, 100 μM stock solutions of lyophilized crRNA and tracrRNA were prepared using nuclease-free duplex buffer, supplied by IDT, and stored at −20°C until use. The Cas9 enzyme, delivered at a concentration of 10 µg/µl, was diluted 1:10 to a final concentration of 1 µg/µl using nuclease-free Cas9 working buffer (20 mM HEPES, 150 mM KCl, pH 7.5) and stored at −20°C until use.

To delete the entire coding sequence of the *pksP* gene, a dual Cas9-gRNA system was implemented. To do this, two separate crRNAs were designed to target selected protospacer sequences in the 5′ UTR and 3′ UTR of the *pksP* gene. These crRNAs were designated cr5′pksP and cr3′pksP, respectively (Table 1). Selection of the protospacer sequences is described in Results (Fig. 2). For generation of the gRNA, each crRNA was separately hybridized to the tracrRNA by mixing equal molar amounts of crRNA and tracrRNA in nuclease-free duplex buffer to a final concentration of 33 μM (Fig. 1). The two mixtures were boiled at 95°C for 5 min and then cooled to room temperature (20 to 25°C) for 10 to 15 min. The resulting gRNAs were stored on ice for up to 5 h preceding transformation or at −20°C for long-term storage. For generation of the Cas9 RNP complexes, 1.5 µl of each gRNA was separately combined with 0.75 µl of Cas9 (1 µg/µl) and 11 µl of nuclease-free Cas9 working buffer in a sterile 1.5-ml tube (final volume, 13.25 µl). These mixtures were incubated at room temperature for 5 min to allow for the formation of dual RNP complexes (Fig. 1). The two RNP reaction mixtures were mixed to a final volume of 26.5 µl and then used for *A. fumigatus* protoplast transformation, as described below.

### Transformation of *A. fumigatus*.

Transformation of *A. fumigatus* protoplasts was carried out according to a standard protocol (56), with minor modifications. Briefly, conidia were inoculated into 100 to 150 ml of liquid YG (5 g/liter yeast extract and 20 g/liter d-glucose) at a final concentration of 1 × 10^6^ conidia/ml and cultivated for 16 h at 30°C with shaking at 250 rpm. Mycelia were harvested by filtration, washed with sterile water, resuspended in protoplasting buffer (5% [wt/vol] VinoTastePro lytic enzyme mix, 1.2 M MgSO_4_⋅7H_2_O, 10 mM sodium phosphate buffer, pH 6.5), and incubated at 32°C with shaking at 75 rpm until protoplasts were produced (approximately 2 to 5 h, depending on the strain). Protoplasts were separated from mycelial debris by overlaying the protoplast mixture with trapping buffer (0.6 M sorbitol, 100 mM Tris-HCl, pH 7.0) and centrifuging for 15 min at 3,500 rpm and 4°C. The protoplast layer was transferred to a new tube, washed with 3 volumes of STC buffer (1.2 M sorbitol, 7.55 mM CaCl_2_⋅H_2_O, 10 mM Tris-HCl, pH 7.5), and centrifuged for 10 min at 3,500 rpm and 4°C. The protoplast pellet was then resuspended in STC buffer to a final concentration of 5 × 10^7^ protoplasts/ml. To proceed with Cas9-mediated transformation, 200 µl of protoplasts was transferred to a sterile 15-ml tube containing the full 26.5-µl reaction mixture of the dual RNPs, prepared as described above (Fig. 1). Immediately, 2 or 10 µg of the purified repair template (described above) and 25 µl of polyethylene glycol (PEG)-CaCl_2_ buffer (60% [wt/vol] PEG 3350, 50 mM CaCl_2_⋅H_2_O, 450 mM Tris-HCl, pH 7.5) were added, and the mixture was incubated on ice for 50 min. Afterward, 1.25 ml PEG-CaCl_2_ buffer was added, and the mixture was incubated at room temperature for 20 min. Subsequently, the mixture was diluted to a total volume of 3 ml with STC buffer and plated on SMM agar plates (GMM supplemented with 1.2 M sorbitol and 1.5% [wt/vol] agar). The plates were incubated overnight at room temperature to allow regeneration of the fungal cell wall. Finally, all transformation plates were overlaid with SMM top agar (GMM supplemented with 1.2 M sorbitol and 0.7% [wt/vol] agar) containing hygromycin (final concentration of 150 µg/ml), and the plates were incubated at 37°C for 3 days.

### Southern blot analysis of Δ*pksP* mutants.

A *pksP*-specific probe was amplified from genomic DNA of Af293 by using the primer set pksP-Probe-Forward and pksP-Probe-Reverse (Table 1). The resulting 504-bp PCR fragment was biotinylated with the North2South biotin random primer labeling kit (Thermo Scientific), according to the manufacturer’s protocols. The genomic DNA of 6 arbitrarily selected white colonies, which were transformed with 2 µg of the 35-bp-flanked HygR repair template, was isolated using a standard phenol-chloroform extraction protocol. Following quantification by a NanoDrop spectrophotometer and integrity verification by agarose gel electrophoresis, the genomic DNA was digested overnight at 37°C using the restriction enzyme XhoI and was separated on an agarose gel before being transferred to a Biodyne B modified nylon membrane (Thermo Scientific). Membrane-bound, digested genomic DNA was hybridized with the biotinylated probe and then labeled with horseradish peroxidase-conjugated streptavidin (Thermo Scientific). Finally, the membrane was visualized by using the North2South chemiluminescent hybridization and detection kit (Thermo Scientific).

### Sequencing of clinical isolate DI15-102.

The genomic DNA of the clinical isolate DI15-102 was extracted using a standard phenol-chloroform extraction protocol. For each of the *pksP* dual crRNAs, a 300-bp fragment that spans 140 bp upstream and downstream of the crRNA binding site was PCR amplified. The primer pair pksP(p)-159-For and pksP-141-Rev was used to amplify the 300-bp region flanking the binding site of *pksP* 5′ crRNA. Similarly, pksP-6521-For and pksP(t)-159-Rev were used to amplify the 300-bp region flanking the binding site of *pksP* 3′ crRNA. Both fragments were purified using the GeneJET gel extraction kit (Thermo Scientific) and eluted with nuclease-free water. The purified DNA fragments were mixed with the sequencing primers Seq-pksP(p)-126-For and Seq-pksP-6606-For, which bind 126 bases and 53 bases upstream of *pksP* start and stop codons, respectively. Mixtures were sequenced by Genewiz Inc. using Sanger sequencing technology.
